# Cosmetic Medical Tourists’ Use of Online Support Communities: Sharing Information, Reciprocity, and Enduring Relationships

**DOI:** 10.1177/10497323231219939

**Published:** 2024-01-16

**Authors:** Rowena Forsyth, Tushar Prasad

**Affiliations:** 1 Cyberpsychology Research Group, Biomedical Informatics and Digital Health Theme, School of Medical Sciences, Faculty of Medicine and Health, 4334 The University of Sydney, Camperdown, NSW, Australia

**Keywords:** cosmetic medical tourism, online support communities, cosmetic surgery, social media, digital health

## Abstract

Cosmetic procedures are amongst the most popular procedures sought after by medical tourists. Cosmetic medical tourists utilise numerous sources of information when planning their trips including, where available, discussing their decision with previous medical tourists. Current research on online support communities has investigated the interactions of patients with various health conditions with online support; however, limited research exists on cosmetic medical tourists’ participation in online support communities. Here we report findings from our qualitative interview study of Australian cosmetic medical tourists. We found that many of our participants experienced stigma regarding their intention to receive cosmetic procedures and to travel overseas from within their local social networks. Participating in online communities (Facebook groups) enabled them to access information and support from other cosmetic medical tourists. Through using public posting and messaging functionality of the communities, they performed two distinct roles in the groups that parallel the temporal transitions of their journeys: they were *information and support seekers* pre-surgery and *information and support providers* post-surgery. The reciprocity they practiced in the *provider* role occurred due to their desire to ‘pay forward’ the support they had received from others pre-surgery. This role was performed as a collective, community-based reciprocity rather than a direct mutual exchange. Some participants also transitioned their online relationships into enduing offline friendships demonstrating how online interactions may become enmeshed with broader social networks.

## Introduction

### Cosmetic Medical Tourism

Medical tourists have emerged from a long-standing tradition globally of people travelling away from their home to access health interventions (Bruce, 2013; Frost & Laing, 2016). Medical tourists access a broad spectrum of specialties including cardiology, reproductive medicine, and dentistry (Lunt et al., 2011) which are commonly organised and paid for privately (Hopkins et al., 2010; Turner, 2013). Cosmetic procedures are the most common type of medical tourist procedures, giving rise to the concept of cosmetic medical tourism which Holliday and colleagues (2019, p. 3) define as “travel to access procedures that enhance appearance.” The most common cosmetic procedures performed abroad include breast augmentation, liposuction, and blepharoplasty (International Society of Aesthetic Plastic Surgery, 2017).

Definitive numbers of medical tourists are difficult to ascertain (Connell, 2011, 2013; Horsfall & Lunt, 2015); however, it is estimated that medical tourism is available in more than 40 countries internationally (BusinessWire, 2021). There were an estimated five million medical tourists globally in 2015 (Horsfall & Lunt, 2015) comprising at least twenty-thousand Australians (Holliday et al., 2019).

Individuals consider numerous factors when deciding to travel abroad for medical procedures. Firstly, the cost of medical procedures in developing countries is often significantly lower than the price of medical procedures in developed countries (Horowitz et al., 2007) and the availability of relatively affordable travel packages that include transport, pre- and post-treatment accommodation and health facility, and surgeon bookings (Holliday et al., 2019). Additionally, medical tourists often access procedures that may be unavailable or illegal in their home countries and have shorter waiting times for their procedures in destination countries compared to their home country (Cameron et al., 2014; Hopkins et al., 2010). Cultural familiarity with destinations (Hanefeld et al., 2015) and the opportunity to engage in tourist activities such as touring, shopping, and visiting friends and family (Lovelock & Lovelock, 2018; Musa et al., 2012; Nassab et al., 2010) are also central to cosmetic medical tourists’ decision-making.

We acknowledge the controversies surrounding the terminology of ‘medical tourists’ and the alternative ‘medical traveller’ (Song, 2010; Turner, 2013; Whittaker, 2008). We use the term ‘cosmetic medical tourist’ given that most of the procedures pursued by our participants were relatively low risk and many of our participants stated the possibility of participating in leisure activities during their trips as a motivating factor for their travel (Cohen, 2008; Cook, 2010).

Cosmetic medical tourists (CMTs) may seek information or advice from their family and friends about whether and where to travel abroad for their medical procedure and which medical facilitator to choose but may also ask their family physician for professional help regarding these aspects of their travel (Henson et al., 2015). Australian medical practitioners do not generally support medical tourism due to the widely held belief that the quality of health care in developing countries is of a lower standard compared to developed countries (Australian Society of Plastic Surgeons, 2014). Consequently, cosmetic medical tourists may not solely rely on the opinion of their medical practitioner but conduct their own research through online websites (Johnston et al., 2012). As Holliday and colleagues (2015, p. 301) put it, “It is a truism that without the internet, medical tourism would probably not exist.”

### Online Information, Interaction, and Support

When deciding to travel to receive cosmetic procedures, individuals decide which procedures they will receive, and the facility and the practitioner who will conduct the procedure as well as deciding on the travel aspects of their trip – including transport, accommodation, and destination. Although cosmetic medical tourism is growing, many medical tourists may not have access to a previous medical tourist within their existing social network. For this reason, and due to many medical tourists’ familiarity with exercising consumer choice for health and travel booking, it is often easy for them to research these decisions through the internet. Cosmetic medical tourists conduct research through websites, which includes local and foreign government websites, and cosmetic medical tourism agency websites (Henson et al., 2015) and Facebook pages (Lee et al., 2014) to inform themselves about the logistics of the procedure(s) and the overall journey (Johnston et al., 2012).

Medical tourists seek to normalise their decisions to travel abroad through communicating with previous medical tourists (Cameron et al., 2014), and personal recommendations are central to medical tourists’ choosing their preferred provider (Hanefeld et al., 2015). Given the web-based nature of modern health information seeking and medical tourism, and the unlikelihood that they would have existing support from a previous medical tourist in the offline world, it followed logically that our cosmetic medical tourists would use online support.

Health-related online forums are popular for people seeking both information and support (Tanis, 2008). Online support groups are usually in the form of social media groups, forums, online chat rooms, and blogs (Coulson, 2019). Interactions with online support may be synchronous (live interactions with other people, e.g., live chat rooms) or asynchronous (a static environment where communication evolves, e.g., discussion board) (Coulson, 2019). The common use of smartphones has led to an increase in the use of online support groups in the past two decades (Wright, 2016).

Online support groups have become a significant source of support for people with chronic diseases (Owen et al., 2010; Weinert, 2000). Interactions in online support groups between members do not only provide them with immediate emotional support but members also exchange knowledge and firsthand experiences regarding their medical condition (Kingod et al., 2017). Individuals often remain in groups as it gives them a sense of belonging and connectedness over an extended period (Coulson, 2005; Shoebotham, 2011). Recent reviews (Coulson & Buchanan, 2022; Gerritzen et al., 2022) have found that health-related online peer support groups for people with HIV and AIDS and Parkinson disease have a range of benefits including improved self-worth, improved illness management, greater confidence interacting with practitioners, and access to social support, particularly for people without access to in-person support groups or whose physical limitations restrict their ability to travel. Since members of these online support groups share the same medical condition, it makes it easier to develop friendships as it is thought that illness-related discussions would be burdensome to offline friends and family (Kingod et al., 2017). For members who are geographically dispersed, they can still communicate online with each other, and so online support groups make it easier to maintain these friendships (Coulson, 2005). These groups may also provide a peer community and sense of normality to individuals who are isolated or stigmatised within their existing social networks (Sanger et al., 2019). Considerable boundary work to protect these ‘digital safe havens’ is often undertaken by group administrators to ensure that members are authentic stigmatised individuals (Yeshua-Katz & Hård af Segerstad, 2020). A study of the positive effects of secure online community board participation on people living with HIV’s sense of stigma (Flickinger et al., 2018) found participants who were active posters in the group and those who posted about stigma-related content had the greatest improvement in perceived stigma. Other studies have shown that disclosing perceived stigmatised health issues in online forums can have benefits for help-seeking and interaction with health professionals (Moore et al., 2016) and that participation in stigmatised communities can support subcultural norms that contradict broader social norms and provide sources of social support (Reich, 2020). Within online health communities, members develop high levels of peer trust (Radin, 2006) built through self-disclosure as they share intimacies of their health experiences. These communities are predominantly sites of peer sharing of experiential information which are considered authentic, authoritative, and trustworthy, in contrast to commercially motivated websites or advertising.

Online support communities are crucial to cosmetic medical tourists as they allow CMTs to interact with other cosmetic medical tourists who are geographically disparate, providing social connectedness, access to information, and shared experience as a cosmetic medical tourist. CMTs value connecting with the stories of previous cosmetic tourists as a way of understanding what to expect from their travel and procedures and be familiar with their destination and recovery prior to it occurring (Holliday et al., 2019; Jones et al., 2016). The trustworthiness of peer accounts is contrasted with professional or promotional information from facilities. Connections with previous cosmetic tourists through online communities and video calls (along with embarking on international travel and purchasing post-surgical recovery garments) also contribute to the gaining of momentum towards receiving the cosmetic procedures (McDonald, 2011). These connections may produce a sense of obligation between individuals who have made plans to travel together.

In this publication, we build on this work to present in greater detail not only the information seeking and information provision activities practised within these online groups but also the meanings that these activities had for relationships between group members. We detail how information seeking and information provision were positional roles for our CMTs as they progressed along their journeys. In addition to this previous work, which was limited to only online interactions, we discuss how our CMTs formed relationships in online groups that translated into in-person friendships.

Our cosmetic medical tourists sought information from a range of online and offline sources in deciding on the travel, procedures, and practitioners for their cosmetic procedure journeys. In this article, we focus on the online community and relational aspect of their information seeking to provide a more detailed analysis of how informational aspects of their Facebook group participation were intertwined with the social connectedness they experienced with other group members.

## Methods

Our interest in this project was to understand how Australian cosmetic surgery tourists decided on, experienced, and reflected on their overseas surgery journeys. Our project was framed within a social constructivist paradigm given its focus on the historically, geographically, and socially located meaning-making undertaken by participants during their journeys (Creswell & Poth, 2016). The relational affordance of this perspective where meanings are made in interactions with others was a core interest of the project and further strengthened the use of social constructivism within our project.

We conducted semi-structured in-depth interviews with purposively sampled Australian residents above the age of 18 who had received cosmetic procedures outside of Australia in the past 12 months (retrospective) or who were considering receiving cosmetic procedures abroad (prospective). We chose to use an inclusive definition of cosmetic surgery in order to include as diverse a sample as possible. This led to us recruiting participants who had received or were planning to receive a broad range of procedures including abdominoplasty, brachioplasty, thighplasty, and breast augmentation, as well as removal of trichilemmal cysts and ear pointing.

We advertised the study through a weblink on our university website, on posters at three university campuses, print advertisements in two local newspapers, and posting on five cosmetic medical tourism Facebook groups. The Facebook groups ranged in membership from 500 to over 5000 individuals. Two were location based (Thailand, Philippines), two allowed only women to join (as stated in their group description and rules), and one was run by a medical tourism travel agency. The advertisements included a URL link or QR code which led participants to an online REDCap survey. This survey gave participants access to the participant information statement, asked them to confirm they fulfilled the inclusion criteria, and allowed them to submit their contact details to indicate their interest in participating in an interview. Once participants completed the online survey, they were contacted via phone call to arrange an interview and were emailed the participant information statement and consent form. All participants completed written informed consent prior to commencing the interview.

We conducted eight interviews between January and August 2019. The participants ranged in age from 21 to 58, four lived in New South Wales (NSW), three lived in Victoria (VIC), and one lived in Queensland (QLD). Two participants identified as male and six as female. Five interviews were conducted via phone and three in person. The interviews lasted 35–60 minutes and were audio-recorded.

Participants were classified as prospective only, retrospective only, and both prospective and retrospective. Three participants were classified as prospective only, meaning they were planning on travelling overseas for cosmetic medical procedure(s) and had not previously travelled overseas for a cosmetic procedure. None of our participants were retrospective only, where they had received overseas cosmetic procedures and were not planning any future procedures. Five participants were classified as both prospective and retrospective, meaning they had previously received cosmetic procedure(s) overseas and were planning additional procedures.

The broad focus of our study and inductive approach lent itself to a reflexive thematic analysis (Braun & Clarke, 2006, 2019, 2022). Interviews were conducted by RF and TP. Audio-recordings of the interviews were fully transcribed by one of the authors (TP) and another researcher. The reflexive thematic analysis process unfolded through collaborative research discussions following data familiarisation through transcribing and rereading transcripts. Collaborative research discussions focused on inductively developing and refining codes and themes. This process was also informed by our reading of literature on the topic. The theme of ‘communication and information’ included subthemes for specific information sources which were further coded by the role and meaning of each source. The subtheme ‘social support’ included the subtheme of ‘online support’ – it is this subtheme that is reported in this article. Sematic coding (Braun & Clarke, 2022) was predominantly used according to participants’ stated meaning (e.g. ‘information gathering’ and ‘decision-making’) with some latent coding also included (e.g. ‘limited disclosure’, ‘stigma’, and ‘forming new relationships’). The codes and themes were digitised into NVivo which was used to electronically code all transcripts.

Neither RF nor TP had direct experience of cosmetic procedures or body modifications. RF is an experienced qualitative researcher with expertise in researching health behaviours and information engagement in health care. TP has a clinical background in speech pathology and was new to qualitative research. At the time of the study data collection (2019), minimal empirical research existed on Australian perspectives on cosmetic surgery tourism and we were interested to understand how existing international literature on medical tourism could relate to an Australian population.

All participants names used in this publication are consistent with the participant’s identified gender and are pseudonyms. Additional anonymisation measures were employed including removal of Facebook group names, generalised reporting of participants’ home locations, and not including specific clinic or health professionals’ names.

## Results

Table 1 shows each participant’s membership of online support groups. Six of the eight participants interviewed were members of Facebook groups before receiving their procedure(s). Three participants had chosen to stay in the Facebook groups post-surgery, whereas one had chosen to leave. Four participants had not yet travelled abroad for cosmetic surgery; however, two of these participants expressed their intention to stay in the Facebook group post-surgery.

**Table 1.** Participant Involvement With Online Support Groups.

Participant	Age	Prospective/retrospective	Member of cosmetic surgery Facebook groups	
			Pre-surgery	Post-surgery
Anna	36	Prospective	Yes	N/A
Barbara	57	Prospective and retrospective	Yes	No
Caleb	27	Prospective and retrospective	No	N/A
Dalia	21	Prospective	No	N/A
Eliza	41	Prospective and retrospective	Yes	Yes
Fiona	58	Prospective and retrospective	Yes	Yes
Gloria	52	Prospective and retrospective	Yes	Yes
Hamish	24	Prospective	Yes	N/A

All our participants utilised numerous information resources to research their overseas cosmetic procedure(s) including popular media such as television shows, speaking to family and friends, and websites of medical tourism agencies. Six of our participants discussed accessing online support through Facebook groups, which were chosen based on the geographical location of their cosmetic procedures. Our participants engaged in distinct types of interactions depending on the stage of their progress along the procedure timeline from pre-surgery to post-surgery.

### Limited Offline Disclosure

Many participants stated that they had limited the disclosure of their intention to undergo overseas cosmetic procedure(s) within their local social networks. This included telling only their partners and not telling their friends, colleagues, or adult children about their intention to undergo the procedure(s) and even that they had received the procedure(s), following their return to Australia.

Reasons for this non-disclosure included uncertainty about these people’s reactions and the belief that these reactions would mostly be negative about both the travel and their desire to undergo cosmetic procedures. Some participants had told their family and friends of their plans and had received negative reactions:

[Family and friends said] I was nuts. That “what happens if I would die over there?” “What happens if they botched it?” You know, I was going by myself. You know everyone tried to talk me out of it, except my husband, and he was paying for it, so he didn’t talk me out of it. (Fiona)

Some participants found that their partners or siblings or parents were supportive of them receiving the procedures; however, these local supports did not have prior experience of travelling overseas for cosmetic procedures so were unable to offer advice on logistical or emotional aspects of their trips. Our CMTs desired additional sources of support for their decision-making through social media, specifically Facebook groups, which were readily accessible and easy to use.

Information provision and emotional support were intertwined throughout participants’ descriptions of their interactions with the online communities. Whilst posts may have requested information, the tone of the responses and the language used by other responding group members meant that our participants experienced positive emotional support for their decision to undergo overseas cosmetic procedures.

Participants used different features of the support groups and the social media platform for different purposes. Public posts were used to ask questions about logistical and recovery aspects, whereas private individual and group messages were used to give updates about experiences.

### Public Posts – Asking Questions and Seeing Others’ Results

#### Information Seeking

Participants would publicly post on the discussion page of joined groups, mainly to ask questions to other members who had undergone cosmetic surgery abroad. Anna asked questions regarding travel companies:

I asked the other day it was “how do I make a decision on what company to go with?” And then people have just written under their recommendations on what companies they’ve used. (Anna)

whereas Gloria asked questions regarding the general procedure:

I asked people who had the weight loss surgery and I asked open-ended questions. (Gloria)

While participants submitted public posts to ask questions and comment, they also used online groups to read about members’ experiences pre-surgery and post-surgery:

Just to see how people would feel … how they prepared themselves, what to expect after surgery. (Gloria)

Just to hear other stories, see pictures of surgeons’ work, see what they’ve done. (Anna)

Some members within these groups experienced complications with their surgery and posted their experiences on these online groups. Reading these had instigated hesitation for Gloria:

I was on a Facebook page before and people were complaining about chest pain … and that put me off a little bit. (Gloria)

Caleb also expressed similar concerns to Gloria:

I did a quick search of like you know fears … just to see what people would say for their answers and stuff like that … and a lot of people had the same kind of like fear or hesitation … that I had. (Caleb)

#### Before and After Images

Participants often viewed before and after images posted by other members as a way to compare themselves and imagine how their body would potentially look after surgery. The friendly tone of the groups meant that participants felt encouraged to share information. Seeing images of members who had complications associated with surgery did not provoke fear due to the supportive nature of the group. Anna described how this support helped normalise surgery recovery and identify when people needed additional post-surgery care. She gave the example of a photo of a person’s surgical wound and the advice that group members offered:

If they’ve had the same sort of experience in the past, they’ll say “oh that’s okay” you know. “You’re only day five from surgery, it’ll clear up, mine looked like that for a little while.” Or, “oh no that probably looks a bit yucky. You probably should go down to emergency and have it checked” … it’s very supportive. (Anna)

Hamish discussed how the surgeon was also a member of the Facebook group he was in and so offered follow-up care after seeing photos of his patients’ complications on the group:

There’s people that have had like you know their healing hasn’t been perfect, and that has affected how the ear has resulted but like they’ve posted that to the group and said by the guy [surgeon] himself, he’s gone “come back in and I’m gonna fix that.” (Hamish)

After seeing that other members had positive results with their surgery, this further helped participants finalise their decision to undergo cosmetic surgery abroad:

I know that there was always going to be negative stigma about going overseas, but once I’d seen the results, I was quite happy going overseas to that doctor. (Eliza)

Furthermore, participants felt reassured and gained confidence in their procedures from viewing non-enhanced photos provided by individuals of their own bodies which they distinguished from those used for promotional purposes by the clinics:

I can kinda trust the people that are getting it done more than I can trust one specialist or one person advertising their services … when you actually see the people getting it done and how they’re responding and their results are good and they’re happy with it, then I can trust that and go ok. (Hamish)

### Public Posts – Sharing Experiences and Supporting Others

Participants would comment on public posts if they had relevant knowledge or an opinion about the post. For instance, the administrator in Gloria’s online group would forward posts to her and ask her to contribute a response if she thought that the question asked was within Gloria’s experience:

When questions are asked, Tina who owns the [cosmetic surgery travel] company, she always tags me in the posts, and I give my own feedback. (Gloria)

For participants who had undergone cosmetic surgery abroad, they shared their own before and after images in the online group and wrote about their experience alongside.

I just posted the photo of me 2 hours after the operation that I was up and about. (Gloria)

I posted photos up and “it’s, you know, the best decision I’ve ever made” … this is what happened to me, this is my story. (Fiona)

Additionally, participants would respond to public posts submitted by other members to show support to each other that they may not receive in an offline environment:

These are all people that have done it [had surgery], and we’re there to support you through it, that’s what it’s all about, so forget about the outside world, they haven’t done it. (Fiona)

Fiona was the administrator of one of the Facebook groups and expressed her responsibility to support other members of the group:

The girls [group members] are saying [to other members] “asking the questions you’re asking is nothing that we haven’t all asked ourselves” … it’s just supporting each other through it. (Fiona)

### Private Message

In addition to public posts, some participants also used private messages to share photos and information. Gloria created a sub-group with four other members of the group who were considering undergoing the same surgery as her. Their relationship had remained solely online as three members lived in different states and one lived in another country. Gloria shared photos and messages with her group as she received her procedures:

I took photos of my hotel room, my hospital room where I stayed, a photo of me with the surgeon, so it’s not unfamiliar .… and I said “y’know this is where I am, this is how I’m feeling,” and then the other women said “oh, y’know can you update us on it as well?” … instead of sending them messages individually, I just put it all in a group … There’s four women that I’ve created a chat with ‘cause they said to me “can you keep us updated?” … so once a week I check in with them, see how they’re going, and then I tell them about my week, my weight loss, what went well, what didn’t went well for me. (Gloria)

On the other hand, Barbara was only contacted through private messaging because she had left the online group and was encouraged to re-join:

I put a comment “is this an American site?” And somebody said “yes,” I thought “oh well y’know that explains why I don’t understand what they’re talking about,” so I unjoined and then I got a private message from somebody that said “just because we’re an American site, you can still stay, join again. We’ve got people from all over the world.” And I said to her, “but I don’t understand about some of the things they’re talking about” … the clinics that they were talking about to go and get laser scar removal … I had no idea what they were talking about and I thought well y’know I don’t need to get notifications for all this stuff that I don’t think is relevant for me, so that’s why I left … “go and get laser skin removal treatment in Texas,” I couldn’t give two hoots. (Barbara)

Despite Facebook making contact with other cosmetic tourists overseas as easy as reaching people in Australia, Barbara found that the procedures and destinations discussed in this overseas-based group were irrelevant for her. This indicates that although geographic distances between people are collapsed in the online space, local contextual aspects of the individual’s health care experience were important for establishing the relevance of the online community for individuals.

### Relationships

#### Group Membership

Not only were these online spaces used as a source of readily available information, they were also used as a form of support. Aside from gathering logistical information on “what company to go with,” Anna reported that she intended staying in these groups post-surgery because “I like interacting with people” and that she would share her experiences with prospective cosmetic medical tourists within the group because “it’s just a way to communicate for the next lot of people that need to know experiences.” She recognised that the Facebook groups also serve as a medium through which relationships could be formed: “If someone wanted just to make some friendship group [that] really appealed to me.”

Some participants directly contrasted the positive interactions they had with the members of the online support communities with their experiences of negative perceptions of overseas cosmetic surgery held by popular media and their local social network. They felt that the online group was a place where their decisions were supported and so had a general positive relationship with the online group:

I talked to my [Facebook] group and they’re all going “oh make sure you put pictures up, how exciting, how exciting.” I wouldn’t even tell anybody here [at home] that I was having it done. I feel everyone is so negative. (Fiona)

#### Transition of Online Relationships to Offline Relationships

In some cases, the relationships formed within these online groups moved from online to in-person. For instance, Anna had formed a relationship with a woman whom she had met on an online group, which continued into the real world:

[I]t was on a page on Facebook, looking for someone to look after her animal. And I’m like “I can come and do it, I’m not far from your place” … I went and met her and met her animals and I looked after them for her.

On the other hand, participants who lived far away from their fellow group members contacted each other via phone and in-person visits:

Oh, she’s about 4 hours away. She’s on the other side of Melbourne … so we don’t catch up physically, but we catch up on the phone … I’m going to see her in a couple of weeks.

Fiona lived in a different state to her friend who she met through the online group. She stated that they “became really good friends online” and “talked to each other all the time” which subsequently translated into in-person interaction:

I went to Canberra and met one of the girls that had had three operations with this doctor … she was quite happy to show me what he had done, and it actually blew me away – the before and after photos. So, that was probably where I went “yep, this is for me.” She showed me her breasts, she had a tummy tuck, and she also had – she had lost 62 kilos and he had basically reconstructed her ‘cause she had a lot of excess skin and he had basically reconstructed her – she had her legs done, her tummy, her breasts, and her arms had done, and the reason I went to her was because she had three operations with this doctor and she’d been to Manila three times … I’d already made my mind up I was going prior to seeing her … But it was just like, y’know, the icing on the cake?

Evident in the above quote is the intimacy of this relationship: not only did Fiona talk to this woman about her experience of the surgery but that this woman also showed her the results of her surgery on her body. This interaction served to foster trust for Fiona in her intended procedures and surgeon and was a crucial part of her decision-making.

The statement reiterates that multilayered function online support groups offer. Amidst communicating, gathering, and evaluating information to make informed choices, online support groups fostered a sense of camaraderie and community amongst like-minded cosmetic tourists determined to complete their surgical pursuits. This sense of community is further evident in Fiona’s account of how she provided reassurance to a group member who was having second thoughts about her procedure. Arguably, the following statement typifies the function of online Facebook groups for a vast majority of our cosmetic tourists:

These [group members] are all people that have done it and we’re there to support you through it, that’s what it’s all about, so forget about the outside world. They haven’t done it. They don’t know what they’re talking about. Talk to the people who have done it and we’ll help you through it.

#### Enduring Relationships and Future Surgery

As noted above, some participants were quick to develop offline relationships with other group members that sometimes translated into in-person meetings. Four of our five participants who were both retrospective and prospective CMTs maintained their membership of their online group after their initial procedures. For Barbara, her friendship with another group member led to them considering travelling together for future procedures:

[My friend] wants to go back over for a boob lift … We can never sort of come up with a time that suits us both ... I’m ready to go now, I want it done now, I want it over and done now … now she bought a café well she’s got less time … I will go over with her for a support person most definitely. I’ll go. If she wants a support person, I’ll definitely go over with her. (Barbara)

## Discussion

Our study strengthens evidence for the crucial role that online support communities play in providing cosmetic medical tourists with a sense of social connectedness, access to information, and shared experiences of cosmetic medical tourism. Consistent with previous research (Coulson, 2005; Shoebotham, 2011), our CMTs sought experiential expertise through their participation in online communities and used these online communities for information support, emotional support and esteem support.

### Information and Support Seeker and Information and Support Provider Roles

The results above demonstrate that our cosmetic medical tourists occupied different roles in online communities at different stages of their surgery journeys. The temporal trajectory of their role in the groups paralleled their cosmetic surgery journey as they performed identifiable shifts in their roles from that of *information and support seeker* pre-surgery to *information and support provider* post-surgery.

When occupying a *seeker* role at the beginning of their CMT journey, individuals would join online groups primarily to seek information, and emotional support and esteem support pre-surgery, which was achieved by submitting public posts on the groups’ main page. Other members of the online support group would then comment on these posts, providing emotional support or advice to these prospective CMTs. Our participants based their decision-making about surgeons and cosmetic medical tourism travel agencies on other CMTs’ experiences that were shared in their online communities. Other CMTs were regarded as authoritative and authentic sources, and their stories became a form of experiential expertise. These findings are consistent with other studies of online community use by individuals with irritable bowel syndrome (Coulson, 2005) and people undergoing aesthetic plastic surgery (Montemurro et al., 2015).

Our CMTs transitioned to acting in a *provider* role following their procedures. They assumed this role altruistically because they felt a sense of community and engaged in reciprocity where they provided advice and support to prospective cosmetic medical tourists either through public posts or private messages on the Facebook groups. Consistent with Chang et al. (2018), relational closeness was central to the provision of support for fellow group members. Our participants described ongoing active engagement with their groups throughout their cosmetic surgery journeys. Active engagement with others’ posts through replies has been shown to correlate with the number of replies people send, the number of users they reply to, and the length of replies received in online community groups (Pan et al., 2017). In addition to online interactions, our participants also maintained their relationships through phone calls and in-person meetings. This further supports our consideration of online communities as enmeshed with, rather than separate to, offline face-to-face interactions.

### Intimacy, Reciprocity, and Enduring Relationships

Through accessing online communities, our CMTs found support that was unavailable to them in their existing offline relationships. They experienced stigma from their local social networks regarding their intention to undertake cosmetic procedures overseas. In contrast, participating in online communities where people had a shared experience of being stigmatised for these procedures led to them developing trust and receiving encouragement and support from other CMTs. They developed strong bonds with other CMTs since what they had in common with other online community members was not only a shared experience but a shared *stigmatised* experience. Our study provides novel additional evidence for the way that online platforms can be used as therapeutic support for people experiencing stigma (Reich, 2020; Sanger et al., 2019; Yeshua-Katz & Hård af Segerstad, 2020) through showing how CMTs’ perception of themselves as stigmatised in their existing social networks led them to fill a perceived gap in available support by joining online communities where their intention to receive cosmetic procedures overseas was normalised and supported by other group members.

The potential for online communities to create intimate relationships for CMTs (Holliday et al., 2019; Jones et al., 2016) through developing thick trust between members (Radin, 2006) is also evident in our study. We extend this analysis to suggest that this intimacy was the foundation for some of our CMTs whose relationships translated beyond online spaces into real-world interactions. The intimacy in these in-person interactions was initially based on cosmetic procedures and included sharing of ‘results’ by showing cosmetic procedure outcomes on their bodies or planning future cosmetic surgery travel together. As such, the intimate nature of sharing experiences of their bodies further enhanced the empathy and reciprocity of these relationships. These relationships involved meeting in person and became more conventional friendships. Some of these relationships lasted for extended periods of time and involved considerable travel distances – across cities and to other states. As such, online relationships did not replace in-person relationships but instead fostered new social connections that broadened existing social networks.

The reciprocity practised by our CMTs in these online communities when they acted as *providers* was a collective, community-based reciprocity rather than a direct mutual exchange – the group members they shared their experiences with were not usually the same group members who had provided them with information but instead were the ‘next wave’ of CMTs. The sense of community was felt by being a member of the groups with a common experience – the people they provided information to were people they did not know outside of the Facebook group(s). This reciprocity was experientially based and direct – commenting on other peoples’ specific requests with their own experiences or responding to direct messages rather than producing formal resources or travel guides for a general audience. As such, in the *provider* role, the CMTs acknowledged how important the support they had received from others was and so reciprocated this by ‘paying forward’ their own knowledge and experiences to help others.

Prior to widely available internet and mobile devices, support communities of individuals who shared common health issues were usually co-located. They may have attended the same health service and provided support to other ill people. These online communities enable people to cross international geographical boundaries to share their experiences with people who are not co-located. They share the commonality of their health concern but may experience accessing health services in very different ways. Our study has shown that the commonality of their health issue allows them to form relationships and social bonds with people in contexts different to their own. Our CMTs easily found commonalities of experiences with other members of their CMT specific groups beyond their surgical pursuits which led to sustained friendships.

Although increasing in popularity, travelling overseas for cosmetic surgery is still relatively uncommon in Australia. Coupled with this, our participants’ reluctance to disclose their travel and procedure intentions to people in their local social networks meant that there was a shortfall in their available social support which they actively sought to address through connection with other CMTs online. Whilst it may be the case that a proportion of medical tourists do not engage with online support communities, the significance of internet sources of information for CMTs logically suggests that many medical tourists do seek support online. In this online environment, individuals access support from other people with similar experiences rather than solely relying on testimonials from travel company websites. These online communities for sharing health care journeys are likely to maintain and increase in popularity given the plethora of freely available online platforms and thus warrant further investigation.

Previous research has found that individuals join online communities of peers to access authentic experiential information that contrasts with websites of practitioners or clinics that are more commercially motivated (Holliday et al., 2019; Jones et al., 2016). We suggest that this clear demarcation is in fact more nuanced and complex than previously suggested. In the case of cosmetic surgery communities, including those accessed by our participants, some of the medical travel agencies (who organised packaged trips of travel and procedures) have their own Facebook groups where employees monitor and respond to group posts and often post images of clients’ surgical outcomes. In addition, some of these clinics also recruit former patients to comment on prospective patients’ posts (as was the case in our study). As such, this practice serves to blur the boundaries between the professional (commercial) and personal information provision within these online communities. More specific investigation of the implications of these types of roles and online community contributions for patients’ trust in the authenticity of this information is warranted. Additionally, this exploration could be usefully analysed for how it may influence momentum towards surgery.

Our CMTs all referred to their decision to undertake cosmetic procedures overseas as a long term decision and that their online community interactions helped them to decide on different features of their trips. It was unclear however whether groups’ interactions permitted discussion of hesitation or doubt about proceeding with their trips and surgeries. The groups discussed by our CMTs were usually only positive regarding proceeding with procedures. The minimal negative interactions experienced by a minority of our participants were dealt with by the individual removing themselves from the group or limiting their engagement within the groups posting and responses. A more sustained analysis of possible and actual negative interactions, resistance to positive discourse and criticism of clinics or practitioners in these groups, and the implications they would have for altering individuals’ momentum towards surgery (McDonald, 2011) would be an additional interesting avenue for future research.

### Limitations

We acknowledge that our study had a small number of participants. However, the strength of our sample is in its diversity: the participants ranged in age from 21 to 58, included both males and females, and lived in urban and regional areas in three different states of Australia. In addition to more common cosmetic procedures (such as thighplasty and breast augmentation), it also included less well-known procedures (buttock augmentation, ear pointing, removal of trichilemmal cysts). It is suggested that further research be conducted regarding how medical tourists seeking different types of procedures interact with online support groups, which could also be supplemented by textual analysis of content on different social media platforms.

## Conclusion

Our study contributes to the evidence base for understanding how individuals use online communities for information and emotional support in health care. Our study had a relational focus on the experiences of using groups and how relationships developed through information seeking and information provision.

Cosmetic medical tourists are a group of particular interest for this phenomenon given their use of internet-based resources for sourcing information for their procedures. Our study adds novel insight in that it extends the reach of previous work (Holliday et al., 2019; Jones et al., 2016) beyond the limits of joining the groups immediately prior to travel and completing the surgical recovery. We identified central reasons for joining groups earlier in this temporal trajectory – the absence of connection with previous CMTs in existing social circles and the stigma experienced when disclosing cosmetic surgery travel intentions to existing social contacts. As such, our CMTs joined online communities not just to seek experiential expertise from others but also to fill a gap in their existing support and information sources. Extending beyond the completion of surgical recovery, we have also shown how online connection about cosmetic surgery travel translated into enduing offline friendships. Our CMTs expected to have commonality and shared experiences with the people they met in online groups beyond just that of cosmetic surgery travel.

Their experience of stigma within their local social networks led to them engaging in online communities for support that was otherwise unavailable. These communities were central to cosmetic tourists’ decision-making not only to gather information pertaining to their procedures but also as a means of developing relationships that strengthened their resolve to seek procedures overseas. Their participation in online communities led to the adoption of roles as *information and support seekers* pre-surgery and *information and support providers* post-surgery. This provider role is a reciprocal role, whereby cosmetic medical tourists believe they have the responsibility to ‘pay forward’ the support they experienced in these online groups pre-surgery. This role was performed as a collective, community-based reciprocity rather than a direct mutual exchange. In some cases, relationships formed through these online groups translated into enduring friendships whereby commonalities beyond cosmetic surgery tourism experiences were found.

## References

[bibr1-10497323231219939] Australian Society of Plastic Surgeons . (2014). Media release: Death sparks renewed warnings about cosmetic tourism. https://plasticsurgery.org.au/wp-content/uploads/2014/10/141027-Death-prompts-warning-on-cosmetic-tourism.pdf

[bibr2-10497323231219939] Braun V. Clarke V. (2006). Using thematic analysis in psychology. Qualitative Research in Psychology, 3(2), 77–101. 10.1191/1478088706qp063oa

[bibr3-10497323231219939] Braun V. Clarke V. (2019). Reflecting on reflexive thematic analysis. Qualitative Research in Sport, Exercise and Health, 11(4), 589–597. 10.1080/2159676X.2019.1628806

[bibr4-10497323231219939] Braun V. Clarke V. (2022). Thematic analysis: A practical guide. Sage Publications. 10.53841/bpsqmip.2022.1.33.46

[bibr5-10497323231219939] Bruce D. M. (2013). Sickness, health, tourism and the ever-present threat of death: Nineteenth-century spa and seasonal travel. In Botterill D. Mainil T. Pennings G. (Eds.), Medical tourism and transnational health care (pp. 13–29). Springer. 10.1057/9781137338495_2

[bibr6-10497323231219939] BusinessWire . (2021). Medical tourism market - Global outlook and forecast 2021-2026 with COVID-19 impact analysis - researchandmarkets.com, 23rd September 2021. https://www.businesswire.com/news/home/20210922005737/en/Medical-Tourism-Market---Global-Outlook-Forecast-2021-2026-with-COVID-19-Impact-Analysis---ResearchAndMarkets.com (Accessed 20th October 2021).

[bibr7-10497323231219939] Cameron K. Crooks V. A. Chouinard V. Snyder J. Johnston R. Casey V. (2014). Motivation, justification, normalization: Talk strategies used by Canadian medical tourists regarding their choices to go abroad for hip and knee surgeries. Social Science & Medicine, 106, 93–100. 10.1016/j.socscimed.2014.01.047 24556288

[bibr8-10497323231219939] Chang P. F. Whitlock J. Bazarova N. N. (2018). “To respond or not to respond, that is the question”: The decision-making process of providing social support to distressed posters on Facebook. Social Media + Society, 4(1), 205630511875929. 10.1177/2056305118759290

[bibr9-10497323231219939] Cohen E. (2008). Medical tourism in Thailand. In Cohen E. (Ed.), Explorations in Thai tourism (pp. 225–255). Emerald. 10.1016/S1571-5043(07)00008-2

[bibr10-10497323231219939] Connell J. (2011). Medical tourism. CAB International. 10.1079/9781845936600.0000

[bibr11-10497323231219939] Connell J. (2013). Contemporary medical tourism: Conceptualisation, culture and commodification. Tourism Management, 34, 1–13. 10.1016/j.tourman.2012.05.009

[bibr12-10497323231219939] Cook P. S. (2010). Constructions and experiences of authenticity in medical tourism: The performances of places, spaces, practices, objects and bodies. Tourist Studies, 10(2), 135–153. 10.1177/1468797611403048

[bibr13-10497323231219939] Coulson N. (2005). Receiving social support online: An analysis of a computer-mediated support group for individuals living with irritable bowel syndrome. Cyber Psychology and Behaviour, 8(6), 580–584. 10.1089/cpb.2005.8.580 16332169

[bibr14-10497323231219939] Coulson N. (2019). Online support communities. In Attrill-Smith A. Fullwood C. Keep M. Kuss D. (Eds.), The Oxford handbook of cyberpsychology (1st ed., pp. 1–21). Oxford University Press. 10.1093/oxfordhb/9780198812746.013.16

[bibr15-10497323231219939] Coulson N. S. Buchanan H. (2022). The role of online support groups in helping individuals affected by HIV and AIDS: Scoping review of the literature. Journal of Medical Internet Research, 24(7), Article e27648. 10.2196/27648 35881456 PMC9364165

[bibr16-10497323231219939] Creswell J. W. Poth C. N. (2016). Qualitative inquiry and research design: Choosing among five approaches. Sage Publications.

[bibr17-10497323231219939] Flickinger T. E. DeBolt C. Xie A. Kosmacki A. Grabowski M. Waldman A. L. Reynolds G. Conaway M. Cohn W. F. Ingersoll K. Dillingham R. (2018). Addressing stigma through a virtual community for people living with HIV: A mixed methods study of the PositiveLinks mobile health intervention. AIDS and Behavior, 22(10), 3395–3406. 10.1007/s10461-018-2174-6 29882048 PMC6153974

[bibr18-10497323231219939] Frost W. Laing J. (2016). History of spa tourism. In Smith M. K. Puczkó L. (Eds.), The routledge handbook of health tourism (pp. 9–19). Routledge.

[bibr19-10497323231219939] Gerritzen E. V. Lee A. R. McDermott O. Coulson N. Orrell M. (2022). Online peer support for people with Parkinson Disease: Narrative synthesis systematic review. Journal of Medical Internet Research Aging, 5(3), Article e35425. 10.2196/35425 35896025 PMC9377481

[bibr20-10497323231219939] Hanefeld J. Lunt N. Smith R. Horsfall D. (2015). Why do medical tourists travel to where they do? The role of networks in determining medical travel. Social Science & Medicine, 124, 356–363. 10.1016/j.socscimed.2014.05.016 24976006

[bibr21-10497323231219939] Henson J. Guy B. Dotson M. (2015). Should I stay or should I go?: Motivators, decision factors, and information sources influencing those predisposed to medical tourism. International Journal of Healthcare Management, 8(1), 4–14. 10.1179/2047971914Y.0000000083

[bibr22-10497323231219939] Holliday R. Bell D. Cheung O. Jones M. Probyn E. (2015). Brief encounters: Assembling cosmetic surgery tourism. Social Science & Medicine, 124(1), 298–304. 10.1016/j.socscimed.2014.06.047 24985788

[bibr23-10497323231219939] Holliday R. Jones M. Bell D. (2019). Beautyscapes: Mapping cosmetic surgery tourism. Manchester University Press. 10.7765/9781526134264

[bibr24-10497323231219939] Hopkins L. Labonté R. Runnels V. Packer C. (2010). Medical tourism today: What is the state of existing knowledge? Journal of Public Health Policy, 31(2), 185–198. 10.1057/jphp.2010.10 20535101

[bibr25-10497323231219939] Horowitz M. Rosensweig J. Jones C. (2007). Medical tourism: Globalization of the healthcare marketplace. Medscape General Medicine, 9(4), 33. PMC2234298 18311383

[bibr26-10497323231219939] Horsfall D. Lunt N. (2015). Medical tourism by numbers. In Lunt N. Horsfall D. Hanefeld J. (Eds.), Handbook on medical tourism and patient mobility (pp. 25–36). Edward Elger Publishing. 10.4337/9781783471195.00011

[bibr27-10497323231219939] International Society of Aesthetic Plastic Surgery . (2017). Demand for cosmetic surgery procedures around the world continues to skyrockets - USA, Brazil, Japan, Italy and Mexico ranked in the top five countries. https://www.isaps.org/wp-content/uploads/2017/10/GlobalStatistics.PressRelease2016-1.pdf

[bibr28-10497323231219939] Johnston R. Crooks V. Snyder J. (2012). I didn't even know what I was looking for": A qualitative study of the decision-making processes of Canadian medical tourists. Globalization and Health, 8(23), 23. 10.1186/1744-8603-8-23 22769723 PMC3475067

[bibr29-10497323231219939] Jones M. Bell D. Holliday R. Probyn E. Taylor J. S. (2016). Facebook and facelifts: Communities of cosmetic surgery tourists. In Lean G. Staiff R. (Eds.), Travel and transformation (pp. 189–204). Routledge.

[bibr30-10497323231219939] Kingod N. Cleal B. Wahlberg A. Husted G. (2017). Online peer-to-peer communities in the daily lives of people with chronic illness: A qualitative systematic review. Qualitative Health Research, 27(1), 89–99. 10.1177/1049732316680203 27956659

[bibr31-10497323231219939] Lee H. Wright K. O'Connor M. Wombacher K. (2014). Framing medical tourism: An analysis of persuasive appeals, risks and benefits, and new media features of medical tourism broker websites. Health Communication, 29(7), 637–645. 10.1080/10410236.2013.794412 24138286

[bibr32-10497323231219939] Lovelock B. Lovelock K. (2018). “We had a ball... as long as you kept taking your painkillers” just how much tourism is there in medical tourism? Experiences of the patient tourist, Tourism Management, 69, 145–154. 10.1016/j.tourman.2018.05.015

[bibr33-10497323231219939] Lunt N. Smith R. Exworthy M. Green S. T. Horsfall D. Mannion R. (2011). Medical tourism: Treatments, markets and health system implications: A scoping review. OECD, Directorate for Employment, Labour and Social Affairs.

[bibr35-10497323231219939] McDonald E. (2011). Transnationalism bodies-in-motion: Experiences of momentum in transnational surgery. In Mascia-Lees F. E. (Ed.), A companion to the anthropology of the body and embodiment (pp. 481–503). 10.1002/9781444340488.ch28

[bibr36-10497323231219939] Montemurro P. Porcnik A. Hedén P. Otte M. (2015). The influence of social media and easily accessible online information on the aesthetic plastic surgery practice: Literature review and our own experience. Aesthetic Plastic Surgery, 39(2), 270–277. 10.1007/s00266-015-0454-3 25697277

[bibr37-10497323231219939] Moore D. Ayers S. Drey N. (2016). A thematic analysis of stigma and disclosure for perinatal depression on an online forum. Journal of Medical Internet Research Mental Health, 3(2), Article e18. 10.2196/mental.5611 PMC4909386 27197516

[bibr38-10497323231219939] Musa G. Thirumoorthi T. Doshi D. (2012). Travel behaviour among inbound medical tourists in Kuala Lumpur. Current Issues in Tourism, 15(6), 525–543. 10.1080/13683500.2011.626847

[bibr39-10497323231219939] Nassab R. Hamnett N. Nelson K. Kaur S. Greensill B. Dhital S. Juma A. (2010). Cosmetic tourism: Public opinion and analysis of information and content available on the Internet. Aesthetic Surgery Journal, 30(3), 465–469. 10.1177/1090820X10374104 20601579

[bibr40-10497323231219939] Owen J. Boxley L. Goldstein M. Lee J. Breen N. Rowland J. (2010). Use of health-related online support groups; population data from the California health interview survey complementary and alternative medicine study. Journal of Computer-Mediated Communication, 15(3), 427–446. 10.1111/j.1083-6101.2010.01501.x

[bibr41-10497323231219939] Pan W. Shen C. Feng B. (2017). You get what you give: Understanding reply reciprocity and social capital in online health support forums. Journal of Health Communication, 22(1), 45–52. 10.1080/10810730.2016.1250845 28027009

[bibr42-10497323231219939] Radin P. (2006). “To me, it's my life”: Medical communication, trust, and activism in cyberspace. Social Science & Medicine, 62(3), 591–601. 10.1016/j.socscimed.2005.06.022 16039031

[bibr43-10497323231219939] Reich J. A. (2020). “We are fierce, independent thinkers and intelligent”: Social capital and stigma management among mothers who refuse vaccines. Social Science & Medicine, 257, 112015. 10.1016/j.socscimed.2018.10.027 30442504

[bibr44-10497323231219939] Sanger S. Bath P. A. Bates J. (2019). 'Someone like me': User experiences of the discussion forums of non-12-step alcohol online support groups, June 2019. Addictive Behaviors, 98, 106028. 10.1016/j.addbeh.2019.106028 31302313

[bibr45-10497323231219939] Shoebotham A. (2011). Therapeutic affordances of online support group use in women with endometriosis. Journal of Medical Internet Research, 18(5), Article e109. 10.2196/jmir.5548 PMC4877503 27160641

[bibr46-10497323231219939] Song P. (2010). Biotech pilgrims and the transnational quest for stem cell cures. Medical Anthropology, 29(4), 384–402. 10.1080/01459740.2010.501317 21082484

[bibr47-10497323231219939] Tanis M. (2008). Health-related on-line forums: what's the big attraction? Journal of Health Communication, 13(7), 698–714. 10.1080/10810730802415316 18958781

[bibr48-10497323231219939] Turner L. (2013). Transnational medical travel ethical dimensions of global healthcare. Cambridge Quarterly of Healthcare Ethics, 22(2), 170–180. 10.1017/S0963180112000540 23507179

[bibr49-10497323231219939] Weinert C. (2000). Social support in cyberspace for women with chronic illness. Rehabilitation Nursing, 25(4), 129–135. 10.1002/j.2048-7940.2000.tb01887.x

[bibr50-10497323231219939] Whittaker A. (2008). Pleasure and pain: Medical travel in Asia. Global Public Health, 3(3), 271–290. 10.1080/17441690701463936

[bibr51-10497323231219939] Wright K. (2016). Communication in health-related online social support groups/communities: A review of research on predictors of participation, applications of social support theory, and health outcomes. Review of Communication Research, 4(2), 65–87. 10.12840/issn.2255-4165.2016.04.01.010

[bibr52-10497323231219939] Yeshua-Katz D. Hård af Segerstad Y. (2020). Catch 22: The paradox of social media affordances and stigmatized online support groups. Social Media + Society, 6(4), 205630512098447. 10.1177/2056305120984476

